# Real‐world utility of plasma GFAP and NfL in early‐onset AD and FTD

**DOI:** 10.1002/dad2.70490

**Published:** 2026-09-21

**Authors:** Pratishtha Chatterjee, Svetlana Ivanic, Adam Southon, Claire McCarthy, Sheila K. Patel, Maja Christensen, David Darby, Ashley I. Bush, Scott Ayton, Michelle M. Mielke, Emilio Werden, Amy Brodtmann

**Affiliations:** ^1^ Florey Institute of Neuroscience and Mental Health University of Melbourne Parkville Victoria Australia; ^2^ Department of Neuroscience, School of Translational Medicine Monash University Melbourne Victoria Australia; ^3^ Macquarie Medical School Macquarie University Sydney New South Wales Australia; ^4^ Eastern Health Clinical School Monash University Box Hill Victoria Australia; ^5^ Department of Medicine, Austin Health University of Melbourne Melbourne Victoria Australia; ^6^ Department of Epidemiology and Prevention Wake Forest University School of Medicine Winston‐Salem North Carolina USA

**Keywords:** blood biomarkers, cognitive disorders, early‐onset Alzheimer's disease, early‐onset dementia, frontotemporal dementia, tertiary care setting

## Abstract

**INTRODUCTION:**

Plasma glial fibrillary acidic protein (GFAP) and neurofilament light chain (NfL) are promising blood biomarkers, but their real‐world diagnostic utility in early‐onset dementia is understudied.

**METHODS:**

Plasma GFAP, NfL, and phosphorylated tau (p‐tau)217 were measured using Simoa in 118 patients with cognitive complaints, clinically classified as non‐neurodegenerative conditions (non‐ND; *n* = 52), early‐onset Alzheimer's disease (EOAD; *n* = 39), frontotemporal dementia (FTD; *n* = 20), or other‐neurodegenerative dementia (ND)/ dementia (*n* = 7). P‐tau217 defined Alzheimer's disease pathology.

**RESULTS:**

GFAP was elevated in EOAD compared to non‐ND, FTD, and other‐ND, and distinguished EOAD from non‐ND (area under the curve [95% confidence interval (CI)]: 0.924 [0.871–0.977]) but performed poorly for FTD versus non‐ND (0.636 [0.496–0.775]). NfL was most elevated in FTD and EOAD, distinguishing both (FTD: 0.869 [0.778–0.961]; EOAD: 0.770 [0.673–0.867]) from non‐ND, but less effective for FTD versus EOAD (0.750 [0.597–0.903]). GFAP appeared stable in severe renal impairment, while NfL increased.

**DISCUSSION:**

These findings support integrating GFAP and NfL into clinical dementia assessments, with GFAP offering added diagnostic specificity in cases of severe renal impairment; however, validation in larger cohorts is needed.

## Highlights

- Plasma glial fibrillary acidic protein (pGFAP) is elevated in early‐onset Alzheimer's disease (EOAD) compared to non‐neurodegenerative conditions (non‐ND), frontotemporal dementia (FTD), and other‐neurodegenerative dementia (ND).
- pGFAP distinguished EOAD from non‐ND but showed weak performance for FTD versus non‐ND.
- Plasma neurofilament light chain (pNfL) was higher in FTD than non‐ND and EOAD but poorly distinguished FTD from EOAD.
- pGFAP appeared to be stable in severe renal impairment, while pNFL increased.

## 1 BACKGROUND

Early‐onset dementia, defined by symptom onset before the age of 65 years, presents unique diagnostic challenges due to its clinical heterogeneity, atypical presentations, and overlap among neurodegenerative diseases (NDs).^1^, ^2^ Early‐onset Alzheimer's disease (EOAD) and frontotemporal dementia (FTD) are the two most common types of neurodegenerative early‐onset dementia.^3^, ^4^, ^5^ Timely and accurate diagnosis is critical for appropriate patient management and is becoming increasingly important with the emergence of new disease‐modifying therapies. However, current diagnostic pathways are reliant on specialist clinical assessments, which are subject to delays. In addition, biomarker confirmation with molecular positron emission tomography (PET) imaging may be geographically or financially inaccessible, particularly outside research settings.

In this context, blood biomarkers have gained considerable attention as scalable tools that can improve diagnostic accuracy in dementia. Plasma tau phosphorylated at the threonine‐217 residue (p‐tau217) already demonstrates excellent accuracy in reflecting Alzheimer's disease (AD) pathology.^6^, ^7^, ^8^ Additionally, plasma glial fibrillary acidic protein (GFAP), a marker of astrocyte reactivity, is associated with AD pathology and neuroinflammatory processes.^9^, ^10^, ^11^ Neurofilament light chain (NfL), a marker of axonal damage, is elevated across a range of NDs and has been reported to distinguish these from psychiatric or functional disorders.^12^, ^13^ In line with this, both plasma GFAP and NfL have been reported to be elevated in AD and FTD compared to cognitively unimpaired (CU) individuals.^13^, ^14^, ^15^ GFAP performance may be superior to NfL in distinguishing AD from CU and FTD, with the converse for NfL in distinguishing FTD from CU and AD.^13^, ^14^, ^15^

Most studies of EOAD and FTD have been in highly selected research populations. There are fewer data from representative real‐world cognitive disorders clinical settings, comprising patients with a range of disorders associated with cognitive complaints in people aged < 65 years. Furthermore, most existing biomarker studies have been conducted in controlled research cohorts with strict inclusion criteria and fasted sample collection protocols, which limits their applicability to real‐world clinical settings.

In this study, we evaluated the diagnostic performance of plasma GFAP and NfL, as markers of astrocyte reactivity and neurodegeneration, respectively. These key processes are associated with AD and FTD but are not specific to their core pathologies. We assessed these biomarkers in individuals who presented with cognitive complaints at a tertiary cognitive disorders clinic and had symptom onset before the age of 65 years. Our aim was to evaluate the performance of plasma GFAP and NfL in distinguishing EOAD and FTD from each other, as well as from non‐neurodegenerative conditions (non‐ND) and other‐ND and dementias that are associated with cognitive complaints. We hypothesized that GFAP would be elevated in EOAD compared to non‐ND, FTD, and other‐ND; and that NfL would be elevated in both EOAD and FTD compared to non‐ND.

## 2 METHODS

### 2.1 Participants and diagnostic evaluation

Participants were included from the Neurofilament and Voice Acoustics in Dementia Diagnosis (NAVAIDD) study (ClinicalTrials.gov NCT06339190), approved by the Eastern Health Human Research Ethics Committee (Approval number E21‐006‐72840).^16^ Adult participants with symptom onset prior to age 65 years were included. They presented for diagnostic evaluation for cognitive disorders at the Eastern Health Network in Melbourne, Australia.^17^ This health service region includes a population in which 26% come from non–English‐speaking backgrounds. Written informed consent was obtained from all participants prior to their enrolment.

**RESEARCH IN CONTEXT**

1. **Systematic Review**: We reviewed the literature using PubMed. Plasma glial fibrillary acidic protein (GFAP) and neurofilament light chain (NfL) are promising blood biomarkers for Alzheimer's disease and other neurodegenerative diseases (ND), but their head‐to‐head evaluation for distinguishing early‐onset Alzheimer's disease (EOAD) and frontotemporal dementia (FTD) from individuals with other young‐onset cognitive disorders in a real‐world clinical setting with diverse ethnicity, non‐fasting status, and variable blood collection timing remains underexplored.
2. **Interpretation**: GFAP distinguished EOAD from non‐ND, FTD, and other‐ND, but showed poor discriminative performance in differentiating FTD from non‐ND and other‐ND. NfL distinguished FTD and EOAD from non‐ND but showed an uncertain performance in differentiating FTD from EOAD. NfL, but not GFAP, was elevated in individuals with severely impaired renal function compared to those having relatively preserved renal function.
3. **Future Directions**: Further validation is warranted in studies with a larger other‐ND and other‐dementia sample size. Additional studies are also needed to confirm whether GFAP offers specificity in the context of severe renal impairment.

Clinical diagnoses were established through a multidisciplinary consensus process involving cognitive neurologists, neuropsychologists, speech pathologists, an occupational therapist, and a cognitive nurse consultant. Diagnostic case conferences incorporated detailed clinical histories and informant accounts, alongside review and interpretation of magnetic resonance imaging (MRI); fluorodeoxyglucose PET (FDG PET); and results from neuropsychological, occupational therapy, and speech therapy assessments.^18^

After a consensus meeting of three neurologists, study participants were categorized into one of three groups based on consultation reports, multiple follow‐up letters per patient, and their individual diagnostic assessments: non‐ND (*n* = 52), EOAD (*n* = 39), FTD (*n* = 20), or other‐ND and dementia (*n* = 7). Given the heterogeneity and small number of participants in the other‐ND and dementia group, analyses including this group are only presented in the supporting information (Table S1 and Figures S1–S4).

### 2.2 Blood collection, processing and storage, and measurement of plasma p‐tau217, GFAP, and NfL

Plasma p‐tau217 was measured utilising the ALZpath pTau217 Advantage PLUS Assay kit (Quanterix)^19^ and GFAP and NfL were measured using the Neuro 2‐Plex B Advantage PLUS Assay kit (Quanterix) on the single molecule array (Simoa) HD‐X Analyzer platform, as per the manufacturer's instructions. Calibrators were run in triplicate, quality controls (Control 1 and Control 2) and samples were run in duplicate. Refer to supporting information for percent coefficient of variation (%CV). All kits used to process biomarkers contained the same lot number.

### 2.3 Biological confirmation of AD pathology

Plasma p‐tau217 was used to determine AD pathology status. Abnormal brain amyloid beta (Aβ) status (A+) was defined using a two‐tiered plasma p‐tau217 cut‐off approach: levels < 0.40 pg/mL were considered negative for abnormal Aβ (A–), and levels > 0.63 pg/mL were considered A+. Twelve participants (non‐ND = 7, AD = 3, FTD = 1, other‐ND with dementia = 1) with intermediate p‐tau217 levels were excluded from analyses using biological confirmation of AD pathology. Additionally, levels > 0.64 pg/mL were used to define abnormal tau pathology (T+).^6^ The performance of these plasma p‐tau217 cut‐offs within this cohort have been published previously.^19^ An additional four participants were re‐classified or excluded. Two A–T– participants who were clinically diagnosed with EOAD were re‐classified as non‐AD (e.g., one with likely progressive subcortical gliosis given extensive white matter changes). Two participants who were clinically diagnosed as non‐ND were A+T+ with estimated glomerular filtration rate (eGFR) < 30 mL/min/1.73 m^2^ (end stage renal failure) and were excluded from subsequent analyses on the grounds that plasma protein levels would be uninterpretable given the severity of renal impairment. Given that this is a clinical cohort and most participants did not have amyloid PET or cerebrospinal fluid (CSF) biomarkers, plasma p‐tau217 was used as a surrogate for amyloid PET to perform confirmatory analyses in this subset: 34 A+T+ EOAD and 43 A–T– non‐ND, 19 A–T– FTD, 8 A–T– other‐ND and dementia.

### 2.4 Statistical analysis

Descriptive statistics, including means and standard deviations, were calculated for each group with comparisons using Kruskal–Wallis tests for continuous variables with non‐parametric distributions, general linear models for continuous variables with parametric distributions, and chi‐squared tests or Fisher–Freeman–Halton exact test for categorical variables. General linear models used to compare plasma GFAP and NfL between groups were also adjusted for covariates age, sex, ethnicity, and eGFR (*n* = 91). Plasma GFAP and NfL were natural log transformed to better approximate normality. Logistic regression with diagnosis or diagnosis in combination with AD pathology as response was used to evaluate predictive models. Receiver operating characteristic (ROC) curves were constructed to determine area under the curves (AUCs). DeLong tests were used to compare AUCs. Spearman correlations were used to assess the association of plasma GFAP and NfL with age in all participants. Mann–Whitney *U* tests or Kruskal–Wallis tests were used to compare plasma GFAP and NfL concentrations between females and males, ethnicities, and eGFR ranges within diagnostic groups, as applicable. All analyses and data visualization were carried out using IBM SPSS (v30), GraphPad Prism (v10.4.1) and R (v4.4.2). *p* < 0.05 was considered statistically significant and all statistical tests were two tailed.

## 3 RESULTS

### 3.1 Cohort characteristics

Participant characteristics are presented in Tables 1 and S1. There were more females in the EOAD group compared to the FTD group (67% vs. 30%, *p* = 0.012) but not to the non‐ND group (48%, *p* > 0.05), and there were no sex differences between the non‐ND and FTD group (*p* > 0.05). There was no significant difference in age, age at symptom onset, and eGFR categories among diagnostic groups (*p* > 0.05). There was a significant difference in the frequency of ethnicity types between the EOAD group and the FTD group (*p* = 0.025) in the current sample set, but not between non‐ND and FTD (*p*  > 0.05) or between non‐ND and EOAD (*p* > 0.05). Mini‐Mental State Examination (MMSE) scores were lower in the EOAD group (mean ± standard deviation [SD]: 20.94 ± 5.81; *p*  = 1.28e‐8) and the FTD group (24.64 ± 5.28; *p* = 0.026) compared to the non‐ND group (27.62 ± 2.85). MMSE scores did not differ between the EOAD group and FTD group (*p* > 0.05).

**TABLE 1:** Participant demographics and plasma GFAP and NfL levels in EOAD, FTD, and non‐ND.

	Total *N*	Non‐ND	EOAD	FTD	*p*
	(111)	(*n* = 52)	(*n* = 39)	(*n* = 20)	
Female (*n* (%))	111 (51%)	25 (48%)	26 (67%)	6 (30%)	**0.024**
Age (years, mean ± SD)	111 (60.80 ± 6.78)	60.40 ± 7.18	61.08 ± 6.21	61.30 ± 7.04	0.841
Age at symptom onset (years, mean ± SD)	111 (54.91 ± 8.81)	53.10 ± 10.87	56.64 ± 6.15	56.25 ± 6.30	0.124
Ethnicity (*n*)	102	*n* = 47	*n* = 36	*N* = 19	**0.038**
O	70	28	31	11	
E	19	12	4	3	
A	8	5	1	2	
PA	1	1	0	0	
SA	4	1	0	3	
eGFR (*n*)	100	*n* = 46	*n* = 37	*n* = 17	0.168
≤30 mL/min/1.73m^2^	4	4	0	0	
31–59 mL/min/1.73m^2^	6	2	4	0	
≥60 mL/min/1.73m^2^	90	40	33	17	
MMSE (mean ± SD)	84	*n* = 39	*n* = 34	*n* = 11	**8.54e‐8**
		27.62 ± 2.85	20.94 ± 5.81	24.64 ± 5.28	
Plasma GFAP (pg/mL, mean ± SD)	111	65.56 ± 35.16	153.29 ± 55.44	97.33 ± 83.44	**6.03e‐13**
Plasma GFAP in A/T status biologically classified groups (pg/mL, mean ± SD)	96	*n* = 43	*n* = 34	*n* = 19	**1.09e‐13**
		61.91 ± 35.64	158.41 ± 56.78	81.29 ± 43.83	
Plasma NfL (pg/mL, mean ± SD)	111	9.72 ± 9.97	13.90 ± 7.01	32.07 ± 22.19	**1.28e‐10**
Plasma NfL in A/T status biologically classified groups (pg/mL, mean ± SD)	96	*n* = 43	*n* = 34	*n* = 19	**6.10e‐13**
		7.55 ± 3.97	14.06 ± 6.88	29.33 ± 19.02	

Notes: Demographics and other factors were compared between participants with non‐ND, EOAD, and FTD. General linear models (GLM) or Kruskal–Wallis tests were used to compare continuous variables, while chi‐squared tests or Fisher–Freeman–Halton exact tests were used to compare categorical variables. Ethnicity was determined by place of birth, first language, and in some instances parents’ place of birth and participants were grouped as Oceanian (O), European (E; comprising British, North‐West, Southern and Eastern European), Asian (A; comprising South‐East Asian, North‐East Asian, and Southern and Central Asian), People of Americas (PA), and Sub‐Saharan African (SA). Not all participants disclosed their ethnicity. eGFR and MMSE data were included if taken within 3 years of date of research blood sample collection. Plasma GFAP and NfL were natural logarithmic transformed to better approximate a normal distribution for GLM. A two‐tiered cut‐off for plasma p‐tau217 concentration > 0.4 and ≤ 0.63 was used to determine A–/+ status and > 0.64 pg/mL was used to determine T+ status. For analyses based on biological classification, EOAD cases with A+/T+ status and non‐ND and FTD cases with A–/T– status were included, to avoid including FTD or non‐ND cases with A/T co‐pathology. *p* < 0.05 was considered significant.

Abbreviations: eGFR, estimated glomerular filtration rate; EOAD, early‐onset Alzheimer's disease; FTD, frontotemporal dementia; GFAP, glial fibrillary acidic protein; MMSE, Mini‐Mental State Examination; NfL, neurofilament light chain; non‐ND, non‐neurodegenerative disease; p‐tau, phosphorylated tau; SD, standard deviation.

### 3.2 Comparison of plasma GFAP in EOAD and FTD to non‐ND

**FIGURE 1:**
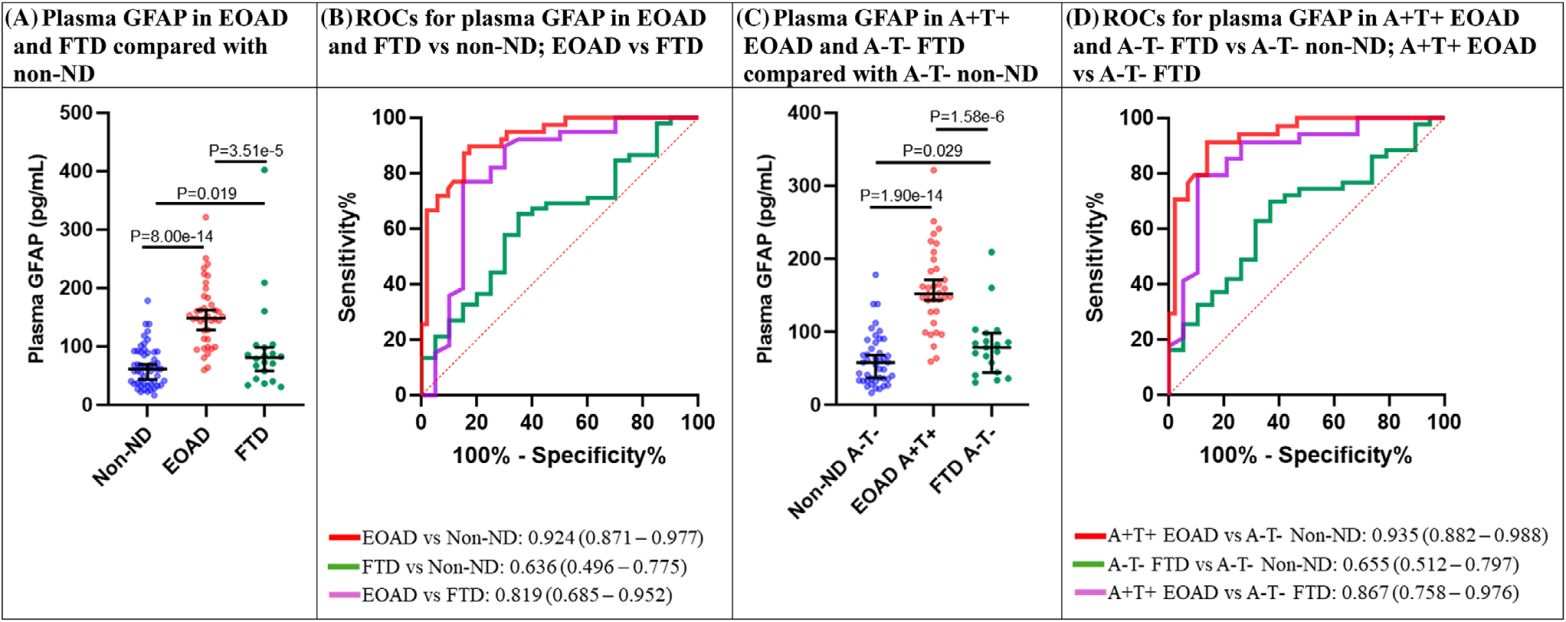
Plasma GFAP in EOAD and FTD compared to non‐ND. A, Plasma GFAP in clinically diagnosed EOAD (*n* = 39) and FTD (*n* = 20) compared to clinically diagnosed non‐ND (*n* = 52), using general linear models. Plasma GFAP values were natural logarithm transformed to ensure model residuals approximated normal distribution; (B) represents ROC curves for EOAD and FTD versus non‐ND, and EOAD versus FTD shown in (A); (C) Plasma GFAP in clinically diagnosed A+T+ EOAD (*n* = 34) and A–T– FTD (*n* = 19) compared to clinically diagnosed A–T– non‐ND (*n* = 43). A–/+ status was defined using a two‐tiered plasma p‐tau217 cut‐off approach, wherein levels < 0.40 pg/mL were considered A– and > 0.63 pg/mL were considered A+, and > 0.64 pg/mL were considered T+. Twelve participants (non‐ND = 7, AD = 3, FTD = 1) within the intermediate zone and two A+T+ non‐ND participants with eGFR < 30 and two A–T– EOAD were excluded for this analysis; (D) represents ROC curves for A+T+ EOAD and A–T– FTD versus A–T– non‐ND, and A+T+ EOAD versus A–T– FTD shown in (C). *p* < 0.05 was considered significant. Error bars in (A) and (C) represent 95% CI, and the line segment represents the median. A, amyloid; CI, confidence interval; EOAD, early‐onset Alzheimer's disease; eGFR, estimated glomerular filtration rate; FTD, frontotemporal dementia; GFAP, glial fibrillary acidic protein; non‐ND, non‐neurodegenerative disease; p‐tau, phosphorylated tau; ROC, receiver operating characteristic; T, tau.

Plasma GFAP was elevated in clinically diagnosed EOAD (mean ± SD: 153.29 ± 55.44) compared to clinically diagnosed non‐ND (65.56 ± 35.16, 8.00e‐14) and clinically diagnosed FTD (97.33 ± 83.44, *p* = 3.51e‐5; Figure 1A). Plasma GFAP was also elevated in clinically diagnosed FTD compared to non‐ND (*p* = 0.019). These observations remained consistent after adjusting for age, sex, ethnicity, and eGFR (*n* = 91; 41 non‐ND, 34 EOAD, 16 FTD). Using ROC curves, plasma GFAP distinguished EOAD from non‐ND (AUC [95% CI]: 0.924 [0.871–0.977]) and FTD (0.819 [0.685–0.952]; Figure 1B). However, plasma GFAP showed poor discriminative performance in differentiating FTD from non‐ND (0.636 [0.496–0.775]). These observations remained consistent when analyses were limited to AD pathology positive (A+T+) EOAD participants (*n* = 34) and AD pathology negative (A–T–) non‐ND (*n* = 43) and A–T– FTD (*n* = 19) participants (Figure 1C,D). Refer to supporting information for cut‐off thresholds. Plasma GFAP was also elevated in EOAD compared to the other‐ND and dementia group (Figure S2).

### 3.3 Comparison of plasma NfL in EOAD and FTD to non‐ND

**FIGURE 2:**
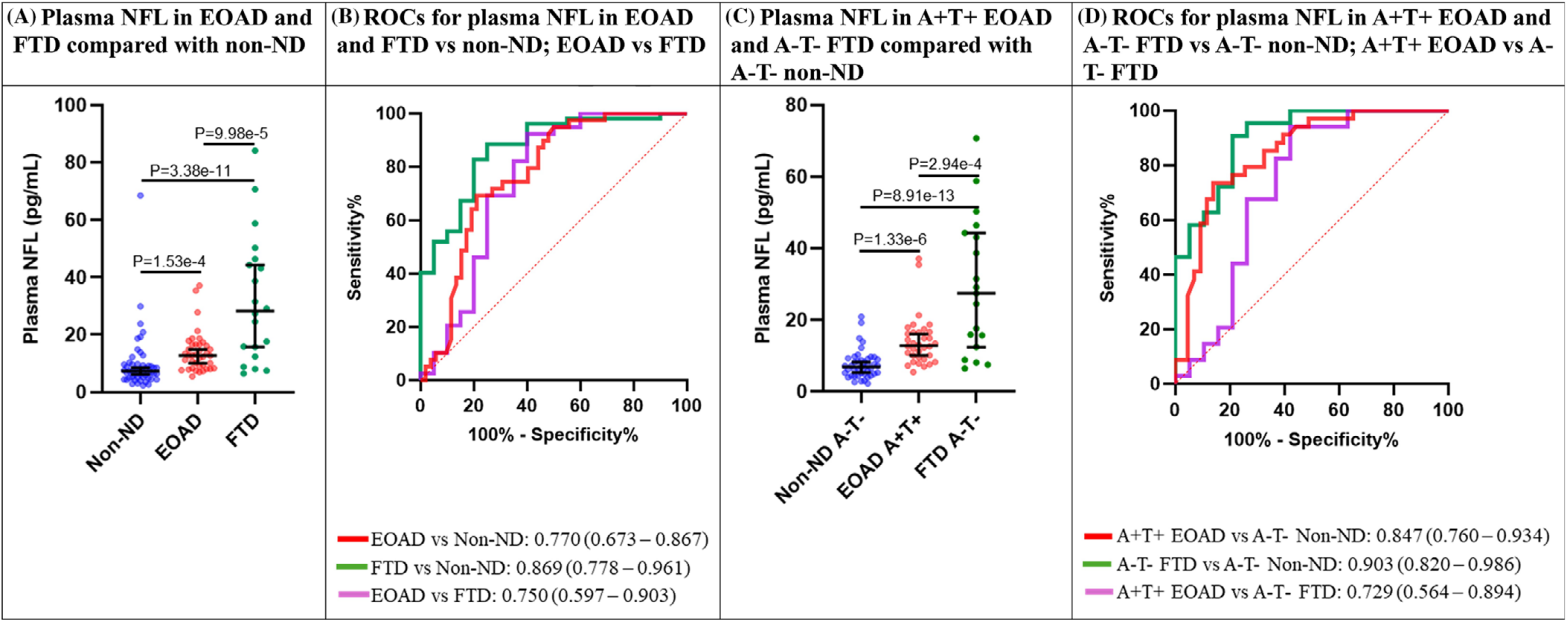
Plasma NfL in EOAD and FTD compared to non‐ND. (A) Plasma NfL in clinically diagnosed EOAD (*n* = 39) and FTD (*n* = 20) compared to clinically diagnosed non‐ND (*n* = 52), using general linear models. Plasma NfL values were natural logarithm transformed to ensure model residuals approximated normal distribution; (B) represents ROC curves for EOAD and FTD versus non‐ND, and EOAD versus FTD shown in (A); (C) Plasma NfL in clinically diagnosed A+T+ EOAD (*n* = 34) and A–T– FTD (*n* = 19) compared to clinically diagnosed A–T– non‐ND (*n* = 43). A–/+ status was defined using a two‐tiered plasma p‐tau217 cut‐off approach, wherein levels < 0.40 pg/mL were considered A– and > 0.63 pg/mL were considered A+, and > 0.64 pg/mL were considered T+. Twelve participants (non‐ND = 7, AD = 3, FTD = 1) within the intermediate zone, two A+T+ non‐ND participants with eGFR < 30, and two A–T– participants with a clinical diagnosis of EOAD which was subsequently re‐classified were excluded for this analysis; (D) represents ROC curves for A+T+ EOAD and A–T– FTD versus A–T– non‐ND, and A+T+ EOAD versus A–T– FTD shown in (C). *p* < 0.05 was considered significant. Error bars in (A) and (C) represent 95% CI, and the line segment represents the median. A, amyloid; CI, confidence interval; EOAD, early‐onset Alzheimer's disease; eGFR, estimated glomerular filtration rate; FTD, frontotemporal dementia; NfL, neurofilament light chain; non‐ND, non‐neurodegenerative disease; p‐tau, phosphorylated tau; ROC, receiver operating characteristic; T, tau.

Plasma NfL was elevated in clinically diagnosed FTD (mean ± SD: 32.07 ± 22.19) compared to clinically diagnosed non‐ND (9.72 ± 9.97, *p* = 3.38e‐11) and clinically diagnosed EOAD (13.90 ± 7.01, *p* = 9.98e‐5; Figure 2A). Plasma NfL was also elevated in clinically diagnosed EOAD compared to non‐ND (*p* = 1.53e‐4). These observations remained consistent after adjusting for age, sex, ethnicity, and eGFR (*n* = 91; 41 non‐ND, 34 EOAD, 16 FTD). Using ROC curves, plasma NfL distinguished FTD from non‐ND (AUC [95% CI]: 0.869 [0.778–0.961]), and EOAD from non‐ND (0.770 [0.673–0.867]; Figure 2B). However, plasma NfL had an uncertain discriminative performance in differentiating FTD from EOAD with a wide CI (0.750 [0.597–0.903]). These observations remained consistent when analyses were limited to AD pathology positive (A+T+) EOAD participants (*n* = 34) and AD pathology negative (A–T–) non‐ND (*n* = 43) and A–T– FTD (*n* = 19) participants (Figure 2C,D). Plasma NfL was also elevated in the FTD group compared to the other‐ND and dementia group (Figure S2).

### 3.4 Comparison between GFAP and NfL in distinguishing between groups

GFAP was superior to NfL in distinguishing EOAD from non‐ND (*p* = 0.019), while NfL was superior to GFAP in distinguishing FTD from non‐ND (*p* = 5.86e‐06). No other significant difference was observed between the performance of GFAP and NfL in distinguishing between groups (Table S2 in supporting information), particularly when AD pathology status was taken in into account.

### 3.5 Association of GFAP and NfL with age, sex, eGFR, and ethnicity

**FIGURE 3:**
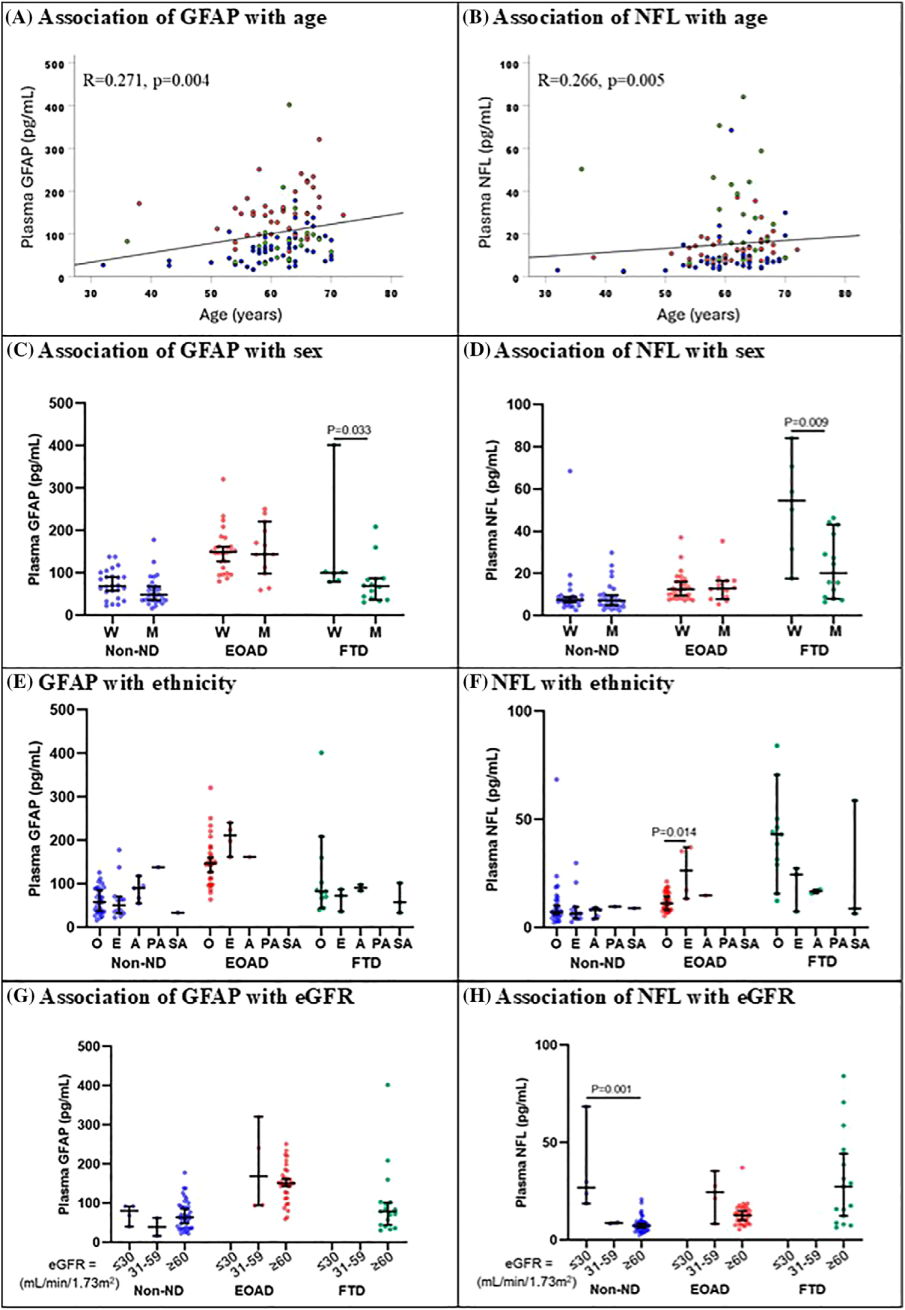
Association of plasma GFAP and NfL with age, sex, ethnicity, and eGFR. A, B, Spearman correlation coefficients were used to assess the association of plasma GFAP and NfL with age in all participants; (C, D) Mann–Whitney *U* tests were used to compare GFAP and NfL between females (W) and males (M) within each diagnostic group; (E, F) Kruskal–Wallis tests were used to compare GFAP and NfL between ethnicities within each diagnostic group and (G, H) between three eGFR ranges, within each diagnostic group. Data on ethnicity and eGFR were only available for 102 and 100 participants, respectively. Blue = Non‐ND, Red = EOAD, Green = FTD. O = Oceanian; E = European comprising British, North‐West, Southern and Eastern European; A = Asian comprising South‐East Asian, North‐East Asian, and Southern and Central Asian; PA = People of Americas; SA = Sub‐Saharan African. Error bars represent 95% CI and line segments represent the median. *p* < 0.05 was considered significant. A, amyloid; CI, confidence interval; EOAD, early‐onset Alzheimer's disease; eGFR, estimated glomerular filtration rate; FTD, frontotemporal dementia; GFAP, glial fibrillary acidic protein; NfL, neurofilament light chain; non‐ND, non‐neurodegenerative disease; p‐tau, phosphorylated tau; ROC, receiver operating characteristic; T, tau.

Both plasma GFAP (*R* = 0.271, *p* = 0.004) and NfL (*R* = 0.266, *p* = 0.005) correlated with age. GFAP and NfL levels did not differ by sex within the non‐ND and EOAD groups (*p* > 0.05). However, among individuals with FTD, females had higher GFAP (*p* = 0.033) and NfL (*p* = 0.009). No significant difference in GFAP and NfL was observed between ethnicities within each diagnostic group (*p* > 0.05), except for lower NfL in Oceanian versus European ethnicity groups in EOAD (*p* = 0.014). No significant difference in GFAP was observed between eGFR groups within diagnostic groups. NfL was significantly elevated in individuals with severely impaired renal function (eGFR ≤ 30 mL/min/1.73m^2^) compared to individuals with eGFR ≥ 60 mL/min/1.73m^2^ in the non‐ND group (*p* = 0.001). While there were no individuals with an eGFR ≤ 30 mL/min/1.73m^2^ in the EOAD group, a trend toward significance was observed for higher NfL in individuals with eGFR between 31 and 59 mL/min/1.73m^2^ compared to individuals with eGFR ≥ 60 mL/min/1.73m^2^ (*p* = 0.070). All participants in the FTD group had eGFR ≥ 60 mL/min/1.73m^2^ (Figure 3).

## 4 DISCUSSION

We demonstrate that plasma GFAP was elevated in individuals with EOAD compared to those with non‐ND (≈ 2.3‐fold higher), FTD (≈ 1.6‐fold higher), and other‐ND and dementia (≈ 2.3‐fold higher) with a lower bound ROC confidence interval > 0.7, indicating good discriminative ability. Although plasma GFAP was also elevated in FTD compared to non‐ND, the fold change was relatively smaller (≈ 1.5‐fold) than that observed in EOAD versus non‐ND. Furthermore, the ROC confidence interval for plasma GFAP in distinguishing FTD from non‐ND cases ranged from 0.5 to 0.8, indicating unreliable discriminative ability.

We also show that plasma NfL was elevated in FTD compared to non‐ND (≈ 4‐fold higher), EOAD (≈ 2.5‐fold higher), and other‐ND and dementia (≈ 1.7‐fold higher). However, the ROC confidence interval for plasma NfL in distinguishing FTD from EOAD ranged from 0.6 to 0.9, and in distinguishing FTD from other‐ND and dementia ranged from 0.6 to 1, indicating unreliable discriminative ability. This may be because NfL is variably elevated in any neurodegenerative disease. Therefore, it does not reliably distinguish between FTD and other ND, even though mean NfL levels are higher in FTD compared to the other ND present in this current real‐world sample set. Although NfL was also higher in EOAD compared to non‐ND, the fold change was relatively smaller (≈ 1.4‐fold higher) than that observed in FTD versus non‐ND; however, the lower bound ROC confidence interval in distinguishing EOAD from non‐ND was > 0.7. We note that while NfL in FTD groups was significantly elevated, the scatter of raw data points in Figure 2C was variable, presenting a limitation in the current study. This could be explained by previous research from our laboratory which demonstrated that NfL differed between FTD syndromes and slowly progressive subtypes, and higher NfL levels discriminated people with FTD syndromes and those without neurodegeneration.^17^ Understanding the clinical phenotype of patients, including their degree of progression, is therefore important for clinical interpretation of NfL levels.

These observations were validated in a subset of study participants classified as either positive or negative for AD pathology. This subset included EOAD participants who were positive for AD pathology biomarkers, as well as non‐ND, FTD, other‐ND, and dementia participants who were negative for AD pathology biomarkers to eliminate the influence of the presence of AD co‐pathology.

In addition to identifying a cut‐off value for GFAP in distinguishing EOAD from non‐ND and other‐ND (GFAP ≥ 95.6 pg/mL), we also investigated a cut‐off value for NfL in distinguishing ND from non‐ND (NfL ≥ 9.88 pg/mL) in individuals with early‐onset cognitive disorders given that NfL distinguished FTD and EOAD from non‐ND. Among individuals classified as either AD pathology‐positive or ‐negative (*n* = 96), six non‐ND cases exhibited elevated levels of GFAP and/or NfL. Of these, four showed isolated elevations in either GFAP or NfL, while two demonstrated concurrent elevations in both biomarkers. Notably, one individual with alcohol‐related brain injury had the highest GFAP (178 pg/mL) and NfL (20.9 pg/mL) concentrations observed in the non‐ND group. These false positive cases may suggest that elevations in both GFAP and NfL are not always specific to ND‐related astrocyte reactivity or axonal damage but may also occur in the context of non‐neurodegenerative insults/injury to the brain. Further, false negatives within the EOAD group for GFAP, and ND group for NfL, may also suggest that some individuals have atypical or less pronounced glial or axonal pathology, potentially reflecting underlying biological heterogeneity within these disease groups.

Growing evidence indicates that elevated blood GFAP in AD reflects Aβ‐associated neuroinflammatory processes.^11^, ^20^, ^21^, ^22^ As a marker of astrocyte reactivity, GFAP in plasma has been linked to monoamine oxidase‐B PET signal, a complementary astrocytic marker, in brain regions susceptible to early Aβ deposition.^11^ Plasma GFAP levels are elevated in individuals with abnormal cerebral Aβ across the AD continuum and can differentiate CU A– individuals from A+ individuals within the preclinical, prodromal, and dementia stages of AD with diagnostic accuracies ranging from 70% to 83% and AUCs ranging 75% to 90%.^9^, ^10^, ^23^, ^24^ Furthermore, plasma GFAP levels correlate with brain Aβ burden and clinical severity, underscoring its relevance as a disease monitoring biomarker.^25^ Notably, using autosomal dominant AD, plasma GFAP could be shown to be elevated approximately a decade before symptom onset, after Aβ deposition but preceding neurodegeneration and cognitive decline, highlighting its potential as an early indicator of pathophysiological change.^25^ Additionally, some of the most compelling evidence that elevated GFAP in AD is Aβ associated comes from an anti‐amyloid trial, in which plasma GFAP levels were lowered in individuals receiving the brain Aβ‐clearing drug, lecanemab, compared to placebo.^26^ However, given that astrocyte reactivity is not specific to AD, it is important to note that GFAP elevations are not exclusive to AD, having also been observed in other ND such as FTD syndromes and dementia with Lewy bodies.^27^, ^28^, ^29^

Elevated plasma NfL in neurodegenerative conditions reflects axonal injury. Unlike plasma GFAP, plasma NfL, as a non‐specific neurodegeneration marker, is not specifically associated with Aβ pathology. It is only quantifiably elevated in the prodromal and dementia stages of the AD continuum, but not in CU A+ preclinical AD.^23^, ^30^ Accordingly, the Aβ pathology–targeting drug, lecanemab, unlike GFAP, did not directly influence plasma NfL levels in response to brain Aβ clearance.^26^ Plasma NfL correlates with clinical severity and cognitive impairment in AD;^31^ however, NfL elevations are not unique to AD and have also been reported in other disorders such as FTD syndromes, multiple system atrophy, and dementia with Lewy bodies, where they similarly reflect the extent of neuronal injury.^12^, ^32^ Furthermore, higher NfL levels in FTD compared to sporadic EOAD may reflect more rapid disease progression typically seen in FTD.^33^, ^34^

Plasma biomarkers for AD are affected by renal impairment (estimated by eGFR).^35^, ^36^, ^37^ In the current study, we observed that plasma NfL, but not plasma GFAP, was elevated in individuals with severely impaired renal function (eGFR ≤ 30 mL/min/1.73 m^2^) compared to those with relatively preserved renal function (eGFR ≥ 60 mL/min/1.73 m^2^) in the non‐ND group. It might be surprising to observe that four participants in the non‐ND group had eGFR ≤ 30 mL/min/1.73 m^2^, but medical comorbidities often lead to cognitive impairments. In addition, people with primary psychiatric disorders such as bipolar affective disorder may have been treated with long‐term lithium, known to cause chronic renal impairment. There were only 11 individuals with eGFR outside normal ranges (< 60 mL/min/1.73 m^2^) reflecting the real‐world nature of the study. While these numbers are low, they provide preliminary insight into the impact of eGFR on NfL and GFAP. This is important to ensure the interpretation of biomarkers remains relevant in individuals with comorbidities. Further studies with greater sample sizes of varying eGFR scores are required to strengthen these conclusions.

Further, plasma p‐tau217, the current leading biomarker reflecting AD pathology,^6^, ^38^ is also elevated in individuals with severely impaired kidney function, which could potentially result in false positive results.^19^ Moreover, the two‐threshold strategy resulted in 10% to 20% of p‐tau217 values falling within the intermediate zone. In such cases, plasma GFAP could serve as a secondary verification marker for individuals with significant renal impairment, and for optimizing participant selection along with p‐tau217 for relevant clinical trials. For instance, among the four non‐ND individuals with severe renal impairment (eGFR ≤ 30 mL/min/1.73 m^2^) in this study, two had p‐tau217 values in the intermediate range (0.4–0.63 pg/mL), while the other two had levels consistent with Aβ positivity (> 0.63 pg/mL). All four exhibited abnormal plasma NfL levels (> 9.88 pg/mL; range: 18–68.5 pg/mL). In contrast, their GFAP levels were all below the abnormal threshold (< 95.6 pg/mL; range: 40.1–92 pg/mL). These findings suggest that GFAP may offer additional specificity in the context of severe renal impairment. However, further studies are warranted to validate this approach. Notably, plasma GFAP has also been reported to show no association with renal function in an independent study, in individuals under 65 years with an eGFR < 60 mL/min/1.73 m^2^.^39^ A similar approach could be applied to patients who exhibit clinical features but have values that fall within intermediate zones or below positive thresholds, using a combination of other biomarkers and/or clinical tests to confirm clinical symptoms of dementia. This strategy improves the diagnostic certainty of biomarkers by reducing misdiagnosis and could guide follow‐up of other clinical tests for inconclusive results.

The strength of this study lies in the head‐to‐head evaluation of plasma GFAP and NfL for distinguishing EOAD and FTD from other individuals with young‐onset cognitive disorders in a real‐world clinical cohort. This type of comparative analysis is largely lacking in the existing literature, in which most studies have examined one condition in isolation. Further, the inclusion of participants with coexisting medical conditions reflects the diagnostic challenges commonly encountered in routine dementia practice. In addition, the study cohort was diverse in terms of ethnicity, fasting status, and blood collection timing. Diagnostic accuracy was strengthened by an initial diagnosis made by an experienced cognitive neurologist supported by multidisciplinary input and review of MRI and FDG PET imaging. Final diagnoses were determined through consensus among three neurologists who were blinded to biomarker results, further increasing diagnostic certainty. We note that all samples were anonymized and analyzed by laboratory staff technicians blinded to participant details. Presented analyses were performed by the authors via diagnostic grouping; therefore, our interpretation is limited on potential bias.

Although imaging or CSF markers of amyloid and tau were not available, plasma p‐tau217 was used to establish amyloid status, given it demonstrated concordance with Aβ PET measures at a level comparable to or exceeding that of CSF biomarkers.^6^, ^40^ We acknowledge the small sample size for the other‐ND and dementia groups. We note that the sample size reflects the distribution typically encountered in clinical practice, particularly early‐ or young‐onset dementia. Furthermore, while plasma GFAP has been reported to be elevated in FTD due to *GRN* mutations,^28^ we were unable to investigate this association as most participants did not consent to genetic testing. It is acknowledged that, while cut‐offs were derived specifically for our laboratory in this study, these thresholds may not be universally applicable due to potential variability between individual Simoa platforms and differences across Quanterix kit version updates. Although cut‐offs for p‐tau217 were derived based on those generated by Ashton et al.,^6^ we have shown these to have excellent performance in confirming amyloid and tau status in our patient cohorts.^19^

In summary, this study provides a comprehensive evaluation of plasma GFAP and NfL for diagnosing EOAD and FTD in a diverse real‐world clinical cohort of individuals with young‐onset cognitive disorders. Plasma GFAP demonstrated good discriminative ability for EOAD, showing significantly elevated levels compared to non‐ND, FTD, and other‐ND and dementia groups, and maintained diagnostic utility when AD pathology/co‐pathology status was accounted for. In contrast, GFAP showed limited utility for distinguishing FTD from non‐ND and other‐ND and dementia. NfL was most elevated in FTD and distinguished both FTD and EOAD from non‐ND conditions, though its ability to differentiate among neurodegenerative groups was limited, consistent with its role as a general marker of axonal injury. This suggests that NfL is best placed to help differentiate ND from non‐ND, but not for disease‐specific diagnosis within the neurodegenerative spectrum. This study also explored biomarker cut‐offs and the influence of comorbidities such as severe renal impairment, for which GFAP showed greater stability than NfL.

Blood‐based biomarkers and diagnostic tools are transforming dementia care, with real‐world clinical studies highlighting their effectiveness in diagnosing late‐onset AD. Overall, our findings highlight the clinical value of GFAP and NfL as complementary plasma biomarkers in the diagnostic workup of young‐onset cognitive disorders and support their integration into dementia assessments. Further validation in larger cohorts and integration with additional biomarkers will enhance their role in precision diagnostics and permit better participant selection in clinical trials.

## CONSENT STATEMENT

Written informed consent was obtained from all participants prior to their enrolment.

## CONFLICT OF INTEREST STATEMENT

Dr. Chatterjee reported receiving grants from the Dementia Australia Research Foundation (DARF), paid to her via her institution. Prof. Bush reports receiving grants from the National Health and Medical Research Council (NHMRC) and the Medical Research Future Fund (MRFF), paid to him via his institution; holding shares in Alterity Ltd, Cogstate Ltd, and Mesoblast Ltd; having a profit‐share interest in Collaborative Medicinal Development Pty Ltd; and receiving lecture fees and travel support from Novo Nordisk P/L, as well as consultancy fees from Biogen. Prof. Ayton reported receiving grants from the NHMRC and MRFF, paid to him via his institution, and personal fees from Eisai Australia and MSD (UK), outside the submitted work. Prof. Brodtmann reported receiving grants from the National Heart Foundation, NHMRC, and MRFF (that supported the submitted work), paid to her via her institution; serving on scientific advisory boards for Eli Lilly, Roche, Novo Nordisk, and Eisai; and acting as an honorary medical adviser for DARF, all outside the submitted work. All other authors report no conflicts of interest. Prof. Mielke has served on scientific advisory boards and/or has consulted for Acadia, Athira, Biogen, Cognito Therapeutics, Eisai, Lilly, Merck, PeerView Institute, Neurogen Biomarking, Novo Nordisk, Roche, Siemens Healthineers, and Sunbird Bio Author disclosures are available in the Supporting Information.

## Supporting information




**Supporting Information**: dad270490‐sup‐0001‐SuppMat.docx

**Supporting Information**: dad270490‐sup‐0002‐ICMJE.pdf
