# Supplementary material for: Real‐world utility of plasma GFAP and NfL in early‐onset AD and FTD
Source: Alzheimers Dement (Amst). 2026 Sep 21;18(3):e70490. doi: 10.1002/dad2.70490 (PMC13593584; doi:10.1002/dad2.70490)
Supplement: Supplementary file 2 [file DAD2-18-e70490-s002.docx]

**Supplementary Figure 1. Participant flowchart.**

**Supplementary Figure 2.** **Plasma GFAP in non-ND, EOAD, FTD compared with other-ND & dementia. (A)** Plasma GFAP in non-ND (n=52), EOAD (n=39) and FTD (n=20) compared with other-ND & dementia (n=7) using general linear models. Plasma GFAP values were natural logarithm transformed to ensure that the model residuals approximated normal distribution; **(B)** represents ROC curves for other-ND & dementia versus non-ND, EOAD and FTD shown in (A); **(C)** Plasma GFAP in clinically diagnosed A-T- other-ND & dementia (n=8) compared with clinically diagnosed A-T- non-ND (n=43), clinically diagnosed A+T+ EOAD (n=34) and A-T- FTD (n=19). A-/+ status was defined using a two-tiered plasma p-tau217 cut-off approach, wherein levels <0.40 pg/mL were considered as A- and >0.63 pg/mL were considered as A+, and >0.64 pg/mL were considered as T+. Twelve participants (other-ND & dementia=1, non-ND=7, AD=3, FTD=1) within the intermediate zone and two A+T+ non-ND & dementia participants with eGFR<30 were excluded for this analysis, and two A-T- EOAD were re-classified as A-T- other-ND & dementia; **(D)** represents ROC curves for A-T- other-ND & dementia versus A-T- non-ND, A+T+ EOAD and A-T- FTD shown in (C). P<0.05 was considered significant. Error bars in (A) and (C) represent 95% CI, and the line segment represents the median. Non-ND: non-neurodegenerative disease; EOAD: early onset Alzheimer’s disease; FTD: frontotemporal dementia; other-ND & dementia: other- neurodegenerative disease and dementia; ROC: receiver operating characteristics.

| **A.** **Plasma GFAP in non-ND, EOAD and FTD compared with other-ND & dementia**   | **B.** **ROCs for plasma GFAP in non-ND, EOAD and FTD vs other-ND & dementia**     | **C. Plasma GFAP in A-T- non-ND, A+T+ EOAD, and A-T- FTD compared with A-T- other-ND & dementia**   | **D. ROCs for plasma GFAP in A-T- non-ND, A+T+ EOAD, and A-T- FTD vs A-T- other-ND & dementia**     |
| --- | --- | --- | --- |

**Supplementary Figure 3.** **Plasma NFL in non-ND, EOAD, FTD compared with other-ND & dementia. (A)** Plasma NFL in non-ND (n=52), EOAD (n=39) and FTD (n=20) compared with other-ND & dementia (n=7) using general linear models. Plasma NFL values were natural logarithm transformed to ensure that the model residuals approximated normal distribution; **(B)** represents ROC curves for other-ND & dementia versus non-ND, EOAD and FTD shown in (A); **(C)** Plasma NFL in clinically diagnosed A-T- other-ND & dementia (n=8) compared with clinically diagnosed A-T- non-ND (n=43), clinically diagnosed A+T+ EOAD (n=34) and A-T- FTD (n=19). A-/+ status was defined using a two-tiered plasma p-tau217 cut-off approach, wherein levels <0.40 pg/mL were considered as A- and >0.63 pg/mL were considered as A+, and >0.64 pg/mL were considered as T+. Twelve participants (other-ND & dementia=1, non-ND=7, AD=3, FTD=1) within the intermediate zone and two A+T+ non-ND & dementia participants with eGFR<30 were excluded for this analysis, and two A-T- EOAD were re-classified as A-T- other-ND & dementia; **(D)** represents ROC curves for A-T- other-ND & dementia versus A-T- non-ND, A+T+ EOAD and A-T- FTD shown in (C). P<0.05 was considered significant. Error bars in (A) and (C) represent 95% CI, and the line segment represents the median. Non-ND: non-neurodegenerative disease; EOAD: early onset Alzheimer’s disease; FTD: frontotemporal dementia; other-ND & dementia: other- neurodegenerative disease and dementia; ROC: receiver operating characteristics.

| **A.** **Plasma NFL in non-ND, EOAD and FTD compared with other-ND & dementia**   | **B.** **ROCs for plasma NFL in non-ND, EOAD and FTD vs other-ND & dementia**     | **C. Plasma NFL in A-T- non-ND, A+T+ EOAD, and A-T- FTD compared with A-T- other-ND & dementia**   | **D. ROCs for plasma NFL in A-T- non-ND, A+T+ EOAD, and A-T- FTD vs A-T- other-ND & dementia**     |
| --- | --- | --- | --- |

**Supplementary Figure 4. Plasma GFAP and NFL in distinguishing Non-ND from ND. (A, C)** Elevated plasma GFAP and NFL in clinically diagnosed non-ND (n=66) compared with ND (n=52) using general linear models. Plasma GFAP and NFL values were natural logarithm transformed to ensure that the model residuals approximated normal distribution; **(B, D)** represent ROC curves distinguishing non-ND from ND shown in (A, C). P<0.05 was considered significant. Error bars in (A) and (C) represent 95% CI, and the line segment represents the median. Non-ND: non-neurodegenerative disease; ND: neurodegenerative disease; ROC: receiver operating characteristics. One participant with vascular dementia was included in the ND group, and while vascular dementia is not typically defined as a neurodegenerative disease, it is generally associated with progressive neuronal loss and was included in the ND group (marked in green).

| **A. Plasma GFAP in non-ND vs ND**   | **B. ROC for plasma GFAP in non-ND vs ND**    AUC (95% CI): 0.800 (0.722 – 0.878) | **C. Plasma NFL in non-ND vs ND**   | **D. ROC for plasma NFL in non-ND vs ND**    AUC (95% CI): 0.792 (0.710 – 0.875) |
| --- | --- | --- | --- |

**Supplementary Table 1. Participant demographics and plasma GFAP and NFL levels including the Other-dementia group.** Demographics and other factors were compared between participants with non-neurodegenerative diseases (non-ND), early onset AD (EOAD), frontotemporal dementia (FTD) and other ND & dementia. General linear models (GLM) or Kruskal-Wallis tests were used to compare continuous variables, while Chi-square tests or Fisher-Freeman-Halton Exact test were used to compare categorical variables. Ethnicity was determined by place of birth, first language, and in some instances parents’ place of birth and participants were grouped as Oceanian (O), European (E; comprising British, North-West, Southern and Eastern European), Asian (A; comprising South-East Asian, North-East Asian, and Southern and Central Asian), People of Americas (PA) and Sub-Saharan African (SA). eGFR and MMSE data were included if taken within 3 years of date of research blood sample collection, and data for ethnicity, eGFR and MMSE were only available for 109, 107 and 90 participants, respectively. Plasma GFAP and NFL were natural logarithmic transformed to better approximate a normal distribution for GLM. A two-tiered cut-off for plasma p-tau217 concentration >0.4 and ≤0.63 was used to determine A-/+ status and >0.64 pg/mL was used to determine T+ status. For analyses accounting for biological classification, non-ND and other-ND & dementia with A-/T- status and EOAD with A+T+ status were included. EOAD were A-T- were re-classified as other-ND & dementia for analyses accounting for biological classification. eGFR: estimated glomerular filtration rate, MMSE: Mini-Mental State Examination. P<0.05 was considered significant.

| Table 1 | N  (non-ND, EOAD, other-ND) | Non-ND  (n=52) | EOAD  (n=39) | FTD  (n=20) | Other-ND & dementia (n=7) | P |
| --- | --- | --- | --- | --- | --- | --- |
| Females (n (%)) | 118 | 25 (48%) | 26 (67%) | 6 (30%) | 2 (29%) | 0.030 |
| Age (years, mean±SD) | 118 | 60.40±7.18 | 61.08±6.21 | 61.30±7.04 | 64.57±5.35 | 0.496 |
| Age at symptom onset (years, mean±SD) | 118 | 53.10±10.87 | 56.64±6.15 | 56.25±6.30 | 59.71±4.61 | 0.096 |
| Ethnicity (n (O, E, A, PA, SA)) | 109  (47, 36, 19, 7) | 28/12/5/1/1 | 31/4/1/0/0 | 11/3/2/0/3 | 7/0/0/0/0 | 0.074 |
| eGFR (n (≤30/ 31-59/ ≥60 mL/min/1.73m^2^)) | 107  (46, 37, 17, 7) | 4/2/40 | 0/4/33 | 0/0/17 | 0/1/6 | 0.224 |
| MMSE (mean±SD) | 90  (39, 34, 11, 6) | 27.62±2.85 | 20.94±5.81 | 24.64±5.28 | 26.00±2.28 | 2.23e-7 |
| Plasma GFAP (pg/mL, mean±SD) | 118 | 65.56±35.16 | 153.29±55.44 | 97.33±83.44 | 67.26±15.78 | 6.11e-13 |
| Plasma GFAP in A/T status biologically classified groups (pg/mL, mean±SD) | 104  (43, 34, 19, 8) | 61.91±35.64 | 158.41±56.78 | 81.29±43.83 | 78.86±33.46 | 1.71e-13 |
| Plasma NFL (pg/mL, mean±SD) | 118 | 9.72±9.97 | 13.90±7.01 | 32.07±22.19 | 18.60±15.88 | 1.08e-9 |
| Plasma NFL in A/T status biologically classified groups (pg/mL, mean±SD) | 104  (43, 34, 19, 8) | 7.55±3.97 | 14.06±6.88 | 29.33±19.02 | 15.71±12.92 | 7.30e-12 |

**Supplementary Table 2. DeLong’s tests for comparison between GFAP and NFL in distinguishing between groups.**

| **GFAP vs NFL in:** | **P value** | **GFAP vs NFL in:** | **P value** |
| --- | --- | --- | --- |
| A+T+ EOAD vs A-T- non-ND | 0.019 | EOAD vs non-ND | 8.66e-4 |
| A-T- FTD vs A-T- non-ND | 5.86e-06 | FTD vs non-ND | 1.76e-05 |
| A+T+ EOAD vs A-T- FTD | 0.249 | EOAD vs FTD | 0.573 |
| A+T+ EOAD vs A-T- Other-ND | 0.055 | EOAD vs Other-ND | 0.005 |
| A-T- FTD vs A-T- Other-ND | 0.059 | FTD vs Other-ND | 0.305 |
| A-T- non-ND vs A-T- Other-ND | 0.730 | non-ND vs Other-ND | 0.306 |

**Supplementary information**

**Participants**: Inclusion criteria for NAVAIDD are broad: any patient presenting to an Eastern Health network health care service with a cognitive complaint or potential ND can consent for a blood test and speech sample. For this analysis, we included those participants with symptom onset at less than 65 years of age.

Referrals to the study were made following outpatient clinic appointments or during inpatient admission. Blood samples were collected at a time convenient for both participants and their care teams, without requiring fasting, to minimise participant burden.

Sex assigned at birth was ascertained. All participants identified as cisgender. Patient ethnicities were not recorded as part of clinical examination. For this study, ethnicity was determined based on place of birth, first language, and where available, parental place of birth. Participants were then grouped according to the Australian Standard Classification of Cultural and Ethnic Groups; Oceanian (broad group 1), European (broad groups 2 and 3), Asian (broad groups 5-7), Peoples of the Americas (broad group 8), Sub-Saharan African (broad group 9) [18]. Data for estimated glomerular filtration rate (eGFR) as a measure of renal function, and Mini-Mental State Examination (MMSE) as a measure of cognitive function, were included if obtained within 3 years of date of research blood sample collection.

**Measurement of plasma p-tau217, GFAP and NFL**: For p-tau217, the average inter-assay %CV for Control 1 and Control 2 was 1.4% and 1.9%, respectively, and the average intra-assay %CV for Control 1 and Control 2 was 2.5% and 1.3%, respectively. For plasma GFAP, the average inter-assay %CV for Control 1 and Control 2 was 7.2% and 4.3%, respectively, and the average intra-assay %CV for Control 1 and Control 2 was 2% and 3.5%, respectively. For plasma NFL, the average inter-assay %CV for Control 1 and Control 2 was 6.9% and 3.1%, respectively, and the average intra-assay %CV for Control 1 and Control 2 was 3.1% and 4.4%, respectively.

**Cut-off thresholds obtained for plasma GFAP and NFL in differentiating early onset neurodegenerative disorders**: Given that plasma GFAP was elevated in EOAD compared with the non-ND, FTD, and other-ND groups, we determined a cut-off value for plasma GFAP to distinguish individuals with EOAD from those with other early-onset cognitive disorders. A Youden index-based cut-off of 95.6 pg/mL for plasma GFAP was identified to differentiate A+T+ EOAD from A-T- non-ND, A-T- FTD, and A-T- other-ND and dementia groups, yielding a sensitivity of 91.2% and a specificity of 82.9%.

Similarly, given that plasma NFL was elevated in FTD compared with non-ND, we determined a cut-off value for plasma NFL to distinguish individuals with FTD from those with non-ND early onset cognitive disorders. A Youden index-based cut-off of 12.3 pg/mL for plasma NFL was identified to differentiate A-T- FTD from A-T- non-ND, with a sensitivity of 78.9% and a specificity of 90.7%.

In addition, we determined a plasma NFL cut-off to differentiate neurodegenerative from non-ND conditions in individuals with early onset cognitive disorders. A Youden index–based cut-off of 9.88 pg/mL yielded a sensitivity of 72.1% and a specificity of 86%.
